# Infantile Neurodegeneration Results from Mutants of 17β-Hydroxysteroid Dehydrogenase Type 10 Rather Than Aβ-Binding Alcohol Dehydrogenase

**DOI:** 10.3390/ijms24108487

**Published:** 2023-05-09

**Authors:** Xue-Ying He, Carl Dobkin, William Ted Brown, Song-Yu Yang

**Affiliations:** 1 Department of Molecular Biology, NYS Institute for Basic Research in Developmental Disabilities, Staten Island, NY 10314, USA; 2 Department of Human Genetics, NYS Institute for Basic Research in Developmental Disabilities, Staten Island, NY 10314, USA; 3 Central Clinical School, University of Sydney, Sydney 2006, Australia; 4 Ph.D. Program in Biology-Neuroscience, Graduate Center, City University of New York, New York, NY 10016, USA

**Keywords:** neurodegeneration, metabolic disorders, mitochondria, X-linked, mental disability, intellectual disability

## Abstract

Type 10 17β-hydroxysteroid dehydrogenase (17β-HSD10), a homo-tetrameric multifunctional protein with 1044 residues encoded by the *HSD17B10* gene, is necessary for brain cognitive function. Missense mutations result in infantile neurodegeneration, an inborn error in isoleucine metabolism. A 5-methylcytosine hotspot underlying a 388-T transition leads to the HSD10 (p.R130C) mutant to be responsible for approximately half of all cases suffering with this mitochondrial disease. Fewer females suffer with this disease due to X-inactivation. The binding capability of this dehydrogenase to Aβ-peptide may play a role in Alzheimer’s disease, but it appears unrelated to infantile neurodegeneration. Research on this enzyme was complicated by reports of a purported Aβ-peptide-binding alcohol dehydrogenase (ABAD), formerly referred to as endoplasmic-reticulum-associated Aβ-binding protein (ERAB). Reports concerning both ABAD and ERAB in the literature reflect features inconsistent with the known functions of 17β-HSD10. It is clarified here that ERAB is reportedly a longer subunit of 17β-HSD10 (262 residues). 17β-HSD10 exhibits L-3-hydroxyacyl-CoA dehydrogenase activity and is thus also referred to in the literature as short-chain 3-hydorxyacyl-CoA dehydrogenase or type II 3-hydorxyacyl-CoA dehydrogenase. However, 17β-HSD10 is not involved in ketone body metabolism, as reported in the literature for ABAD. Reports in the literature referring to ABAD (i.e., 17β-HSD10) as a *generalized* alcohol dehydrogenase, relying on data underlying ABAD’s activities, were found to be unreproducible. Furthermore, the rediscovery of ABAD/ERAB’s mitochondrial localization did not cite any published research on 17β-HSD10. Clarification of the purported ABAD/ERAB function derived from these reports on ABAD/ERAB may invigorate this research field and encourage new approaches to the understanding and treatment of *HSD17B10*-gene-related disorders. We establish here that infantile neurodegeneration is caused by mutants of 17β-HSD10 but not ABAD, and so we conclude that ABAD represents a misnomer employed in high-impact journals.

## 1. Introduction

The *HSD17B10* gene was first cloned from the human brain in 1997 (GenBank^TM^ accession number: AF037438) and mapped to Xp11.2 [1]. It encodes a mitochondrial multifunctional protein with a molecular weight of 108 kDa, composed of four identical subunit molecules. It plays an essential role in isoleucine as well as neurosteroid metabolism [1,2,3,4,5,6,7,8,9,10,11,12,13,14,15]. The rat mitochondrial homolog [16] and the rat *HSD17B10* gene [17] have also been characterized. Because the first enzymatic function identified for this member of the short-chain dehydrogenase/reductase (SDR) family was an L-3-hydroxyacyl-CoA dehydrogenase activity, it was formerly referred to as short-chain 3-hydroxyacyl-CoA dehydrogenase (SCHAD) or L-3-hydroxyacyl-CoA dehydrogenase type 2 (HADII), having 261 amino acid residues per subunit [1].

Nonetheless, a concurrent report described a 27 kDa protein consisting of 262 residues as being associated with the endoplasmic reticulum (ER) and was thus referred to as endoplasmic-reticulum-associated Aβ-binding protein (ERAB) [18,19]. However, this report of an ERAB was found to be a subunit of SCHAD, which contained errors in its reported sequence as well as an erroneous intracellular localization [3,4,5,6,7,8]. Although it cannot be reasonably disputed that the ERAB researchers were aware of reports in the literature of the sequence and activities of SCHAD (i.e., 17β-HSD10), a SCHAD report [1] was cited as a reference in an ERAB *JBC* article [19] regarding the necessity of a corrigendum was readily apparent. Yet, no corrigendum was ever published, despite the journal *Nature* having a policy that “Classes uncorrected errors as misconduct” [20].

**Figure 1:**
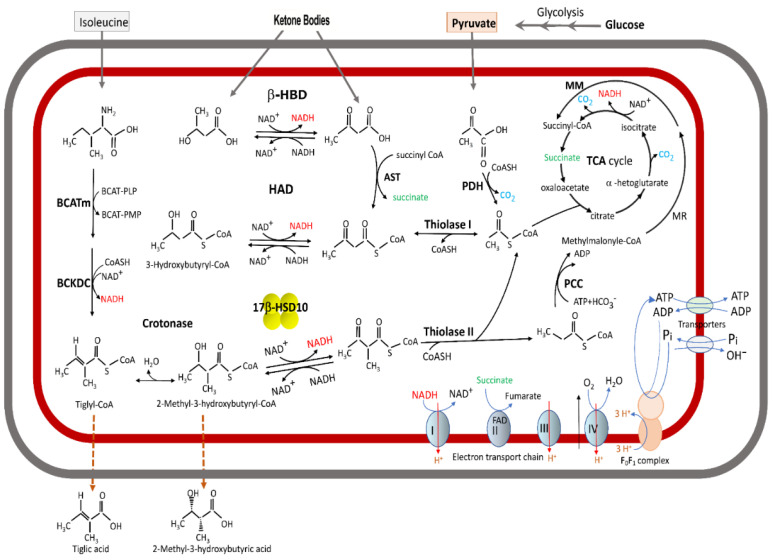
A summary of isoleucine, ketone bodies and glucose metabolism as well as bioenergetics in the brain mitochondria. Abbreviations: AST, acetoacetyl succinyl-CoA transferase; BCATm, branched-chain amino acid transaminase; BCAT-PLP, branched-chain amino acid transaminase pyridoxal 5′-phosphate; BCAT-PMP, branched-chain amino acid transaminase pyridoxamine phosphate; BCKDC, branched-chain α-keto acid dehydrogenase complex; β-HBD, β-hydroxybutyrate dehydrogenase; HAD, 3-hydroxyacyl-CoA dehydrogenase; 17β-HSD10, 17β-hydroxysteroid dehydrogenase type 10; MM, methylmalonyl-CoA mutase; PCC, propionyl-CoA carboxylase; MR, methylmalonyl-CoA racemase; PDH, pyruvate dehydrogenase (reproduced from Figure 1 of Ref. [7]).

Data in the literature establish that SCHAD (i.e., 17β-HSD10) is a new type of 17β-hydroxysteroid dehydrogenase [3,4,5,6]. It is localized in the mitochondria [1,2,3,4,5,8,9,10,11,12,13], although most 17β-hydroxysteroid dehydrogenases are associated with the endoplasmic reticulum [21,22,23,24,25,26,27]. The N-terminal eleven residues of 17β-HSD10/SCHAD form an alpha-helix that serves as a mitochondrial-targeting signal (see Figure 5 of Ref. [5]). This explains why this multifunctional protein is localized in the mitochondria—it is not associated with the endoplasmic reticulum, as the literature concerning ERAB suggests (see Figure 4 of Ref. [18]). Efforts to identify additional mitochondrial-targeting signals in this protein are ongoing. This new 17β-HSD was designated type 10 17β-hydroxysteroid dehydrogenase (17β-HSD10) in an international 17β-HSDs conference [23]. It plays a critical role not only in steroid metabolism (see Figure 1 of Ref. [3]), but also in the metabolism of acyl thioesters [26] (Figure 1). Further, the *SCHAD* gene encoding this multifunctional protein was definitively designated *HSD17B10* by the human genome organization (HUGO) [28,29]. The expression levels of 17β-HSD10 are dependent upon the disease state [30,31].

Because the *HSD17B10* gene product was reported to be a mitochondrial protein [1,2,3,4,5,6,7,8,9,10,11,21,22], moniker ERAB was abandoned by ERAB researchers and thereafter referred to as amyloid beta-peptide-binding alcohol dehydrogenase (ABAD) [32,33] based upon its purported *generalized* alcohol dehydrogenase activity (Table 1), thus removing reference to the localization of ERAB in the endoplasmic reticulum [18,19]. Nonetheless, reports in the literature persisted in asserting that ABAD is associated with the endoplasmic reticulum (see Figure 2C of Ref. [32]). Unsurprisingly, there were reports in the literature concerning relocalized ABAD in the mitochondria [33], but the ABAD reports omitted any reference to the 17β-HSD10/SCHAD literature that had shown its N-terminal-targeting signal resulting in the mitochondrial localization of this protein [1,5,8,9,10,11,21,22]. This series of ABAD/ERAB reports [18,19,32] not only conflicted with the 17β-HSD10 research but also encouraged a trend in the literature to designate the *HSD17B10* gene with various confounding names allegedly related to 17β-HSD10′s individual function. Fifteen alternative names were listed in the Gene ID: 3028, including ABAD, CAMR, ERAB, HCD2, MHBD, HADH2, MRPP2, MRX17, MRX31, SCHAD, MRXS10, SDR5C1, HSD10MD, 17b-HSD10 and DUPXp11.22. This ongoing nomenclature problem [32,33,34,35] directly confounded studies on 17β-HSD10 as well as *HSD17B10*-gene-related disorders. Since 17β-HSD10 is involved in the etiology of human diseases [36,37,38,39,40,41,42,43,44,45,46,47,48,49,50,51,52,53,54,55,56,57,58,59,60,61,62,63,64,65], this is not a trivial issue. This issue deserves to be resolved by a clear and unambiguous correction in the scientific literature [66]. Further studies are desperately needed to firmly eliminate the ongoing confusion in the literature concerning this enzyme to thoroughly resolve potentially harmful inaccuracies. The published corrigenda and/or withdrawal of erroneous reports in the literature may go far in aiding the scientific community to arrive at an accurate consensus concerning this enzyme and, quite likely, benefit the affected patients and their treating physicians.

Although 17β-HSD10 and ABAD were found to have an identical structure [67], they have different reported functions. 17β-HSD10 can bind not only to Aβ [68], but also to other mitochondrial proteins to form RNase P [69], which is not inhibited by Aβ binding [70]. A missense mutation at 17β-HSD10 results in infantile neurodegeneration 17β-HSD10 deficiency [36,37,38,39,40,41,42,43,44,45,46,47,48,49,50,51,52,53,54,55,56,57], for which Aβ peptide levels were undetectable or barely detectable in CSF [71]. This indicated that 17β-HSD10 plays a pathogenetic role in neurodegeneration beyond “the mediation of Aβ neurotoxicity” [18,19,32,33,34,35,72]. Apparently, it is necessary to establish conclusively whether previous reports in the ABAD/ERAB literature concerning its enzymatic function are accurate and reliable or not. Clarification of the alleged differences between 17β-HSD10 and ABAD will go far in helping to restore a proper understanding in the field as well as addressing research integrity issues in this field, which is essential for blazing an accurate and reliable path forward in the search for effective therapeutic targets for *HSD17B10*-gene-related disorders, including HSD10 deficiency or infantile neurodegeneration [36] and Alzheimer’s disease [7].

## 2. Could ABAD Catalyze the Oxidation of D- and L-Hydroxybutyrate at a Similar Rate?

With respect to the catalytic properties of human brain 17β-HSD10/SCHAD, it has been reported [1] that “the substrate stereospecificity of this novel enzyme is opposite to that of acetoacetyl-CoA reductase. The human brain dehydrogenase was found to metabolize only L-3-hydroxyacyl-CoA, but not the D-isomer”. Obviously, the substrate stereospecificity must originate from the orientation of the hydroxy group of β-hydroxybutyrate but not the coenzyme A moiety. Enzymologists would be duly surprised that the oxidation rate of D-3-hydroxybutyrate catalyzed by ABAD/ERAB could be measured spectrophotometrically (see Figure 1 of Ref. [32]). Since the primary structure of ABAD/ERAB is identical to 17β-HSD10/SCHAD, it is highly questioned how ABAD/ERAB could catalyze the oxidation of L-hydroxybutyrate and the D-isomer with almost the same rate (Vmax = ∼4 mU/mg). Furthermore, it was concluded by ABAD/ERAB researchers [19] that “we asked whether it might also catalyze the oxidation of the ketone body D-3-hydroxybutyrate. This was indeed the case, and oxidation proceeded with Km of ∼4.5 mM and Vmax of ∼4 mU/mg”. Assuming for the sake of argument that those reported kinetic constants were not erroneous, it is still questionable whether such an “*ER-associated* NAD^+^-dependent dehydrogenase” (see Figure 2C of Ref. [32]) could play a physiological role in ketone body metabolism; it is well known that most of the cell’s NAD^+^ is sequestered in the mitochondria. Further, life scientists are aware that β-hydroxybutyrate dehydrogenase, β-HBD [73] rather than 17β-HSD10, is involved in ketone body metabolism (see Figure 1). Although data about D-3-hydroxybutyrate supporting the growth of COS cells were provided by Yan et al. [32], such data could not serve as actual evidence for ABAD’s involvement in ketone body metabolism because β-HBD had not yet been knocked out by those investigators. These reports [18,19,32,33,34,35,72] have never been corrected and continue to confound efforts to advance in the physiological role of 17β-HSD10, and thus must be addressed.

## 3. Are Generalized Alcohol Dehydrogenase Activities Reported for ABAD Accurate?

**Figure 2:**
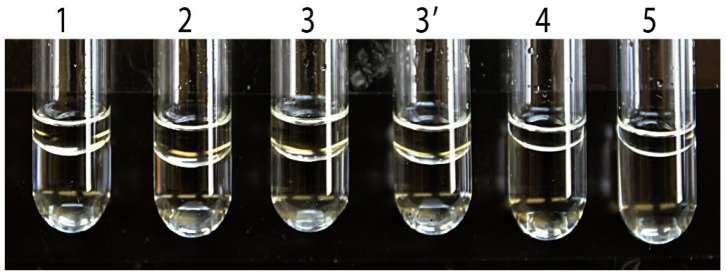
Interface between alcohol and phosphate buffer in generalized alcohol dehydrogenase assay mixtures reported in the literature. Assay mixtures were prepared according to the experimental procedure [19] with 160 mM (-)-2-octanol (tube 1), 210 mM (-)-2-octanol (tube 2), 210 mM (-)-2-octanol with 1% DMSO (tube 3), 210 mM (-)-2-octanol with 1% DMSO mixed with alcohol first (tube 3′), 84 mM (+)-2-octanol (tube 4) and 85 mM (+)-2-octanol (tube 5), respectively. To highlight the interface, the top 1 mL of the assay mixture was transferred to a narrower tube (reproduced from Figure 5 of Ref. [7]).

The oxidation of aliphatic alcohols (C2–C10) catalyzed by ABAD/ERAB was reported by Yan et al. [19] (see Table 1), which is responsible for its misnomer as an Aβ-peptide-binding alcohol dehydrogenase [32,33,34,35,72]. The published experimental procedure in that report is quoted here: ”Alcohol dehydrogenase assays employed ERAB/HADH II (20 µg/mL), a range of alcohol substrate (methanol, ethanol, n-propanol, n-butanol, isobutanol, n-pentanol, (+)-2-octanol, (+)-2-octanol, (−)-2-octanol, and n-decanol; Sigma), and NAD^+^ (7.5 mM) in 22 mM sodium pyrophosphate, 0.3 mM sodium phosphate (pH 8.8). The reaction was run for 2 h at 25 °C, and the absorbance at 340 nm was monitored every 5 min as described above.” When assay mixtures containing (−)-2-octanol or its enantiomers as a substrate with or without 1% DMSO for spectrophotometrically measuring any generalized alcohol dehydrogenase activities were prepared according to the reported experimental procedure (see Table 2), an interface was found between two layers in all of the prepared 2-octanol assay mixtures with or without 1% DMSO (see Figure 2). Thus, it was physically impossible to determine the absorbance changes at 340 nm by using a spectrophotometer, as reported in Ref. [19]. Most of (−)-2-octanol or its enantiomers (the upper layer) remained undissolved in phosphate buffer (the bottom layer) following the addition of 1% DMSO, vortexing for 3 min and then being incubated at 25 °C for 72 h [19], which is due to extremely low solubilities of long-chain alcohols in aqueous media. For example, the solubility of 2-octanol in water is only 8.6 mM at 25 °C [74]. Obviously, the bottom layer contains a rather low concentration of any long-chain alcohol, and so it is impossible to accurately determine the K_m_ value of (−)-2-octanol, previously reported to be as high as 43 ± 9.0 mM (see Table 1), which was derived from the published Figure 2B of Ref. [19]. This establishes, beyond physical doubt, that it would be (and indeed was) impossible for anyone to use 210 mM (−)-2-octanol as a substrate (see the legend of Figure 5B in Ref. [19]) to spectrophotometrically measure any purported generalized alcohol dehydrogenase activity that supports the misnomer Aβ-peptide-binding alcohol dehydrogenase [19,32,33,34,35,72]. Thus, it is not appropriate to consider ABAD/ERAB as an equivalent of 17β-HSD10, e.g., “ABAD/17β-HSD10”, as had been employed in some reports [75,76,77,78,79,80,81].

## 4. The Impact of Aβ-Peptide on the Stereo Structure of ABAD

**Figure 3:**
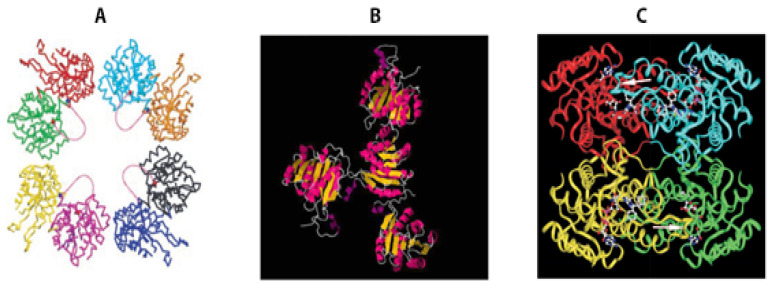
Comparison of the crystal structure images of human Aβ-bound ABAD (**A**,**B**) to that of human 17β-HSD10 (**C**). (**A**) A section of the crystal packing interactions, showing the large solvent channels. Each ABAD molecule is shown in a different color. The ordered ends of the LD loop, residues 94 and 114, are marked as red and blue balls, respectively, and the hypothetical loops are shown in pink as dotted lines [33]; (**B**) PDB: 1SO8 [33] shows only one subunit associated with another three subunits which have no contact with each other; (**C**) PDB: 1U7T [67] shows a close association of four subunits in human HSD10.

The three-dimensional structure of 17β-HSD10 [67] is shown in Figure 3C. According to a report in *Science* [33], this structure would be dramatically altered as Aβ binds to this protein (see Figure 3A,B). Since no electron density of Aβ was reported, it would be highly questionable how the binding of Aβ with ABAD could result in so many different stereo structures (see Figure 3A,B). Although the dissociation constant for Aβ binding to ABAD/ERAB was reported to be approximately 65 nM [19], hundreds-of-fold higher concentrations of Aβ are reportedly required to inhibit the HAD activity of this multifunctional protein [68].

Compared with 17β-HSD10 (see Figure 3C), Aβ-bound ABAD seems to be a very unstable protein (Figure 3B), although it was found [68] that neither RNase P nor methyltransferase activities were specifically affected by the Aβ binding, even though 17β-HSD10 serves as the core of RNase P [69]. Therefore, the accuracy of the three-dimensional structures of Aβ-bound ABAD, previously published in a highly respected journal [33], needs to be verified independently.

## 5. HSD10 Deficiency Is an X-Linked Inherited Metabolic Disease

**Figure 4:**
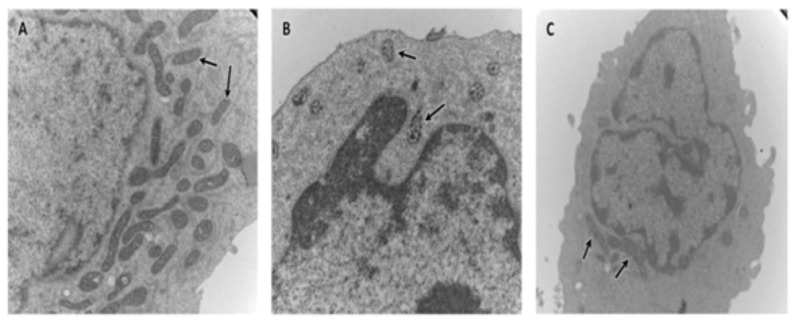
Comparison of the electron-microscopic images of lymphoblastoid cells from patients with an intellectual disability suffering from MRXS10 (**A**) and HSD10 mitochondrial disease (**B**), and normal male control (**C**). The arrows indicate the mitochondria (reproduced from Figure 3 of Ref. [64]).

The *HSD17B10* gene is highly conserved across a large evolutionary distance [75], implying that its gene product 17β-HSD10 plays a vital role [77,78]. It was also found [6] that the post-translational modification of 17β-HSD10 regulates its mitochondrial abundance and its enzymatic activity as well as the formation of RNase P. Missense mutations at the *HSD17B10* gene results in 17β-HSD10 loss-of-function mutants causing HSD10 deficiency [36,37,38,39,40,41,42,43,44,45], formerly referred to as 2-methyl-3-hydroxybutyryl-CoA dehydrogenase (MHBD) deficiency [46,47,48,49,50,51,52,53,54,55,56,57]—an inborn error in isoleucine metabolism. In contrast, a silent mutation R192R alters the quantities but not the quality of 17β-HSD10 caused X-linked mental disability, choreoathetosis and abnormal behavior (MRXS10) [62,63], of which patients showed no blockage in the isoleucine metabolic pathway. HSD10 mitochondrial disease and MRXS10 resulted in different kinds of damage to the mitochondria [64] (see Figure 4). This suggested that a restoration of the OMIM#300220 would be appropriate. Gene duplication of *HSD17B10* also results in mental disability [58]. Since 17β-HSD10 is an essential component of mtRNase P, it was proposed [59,60] that the abnormal processing of mtRNAs is related to the pathogenesis of HSD10 mitochondrial disease.

### 5.1. HSD10 Deficiency Is a Mitochondrial Metabolic Disease

**Figure 5:**
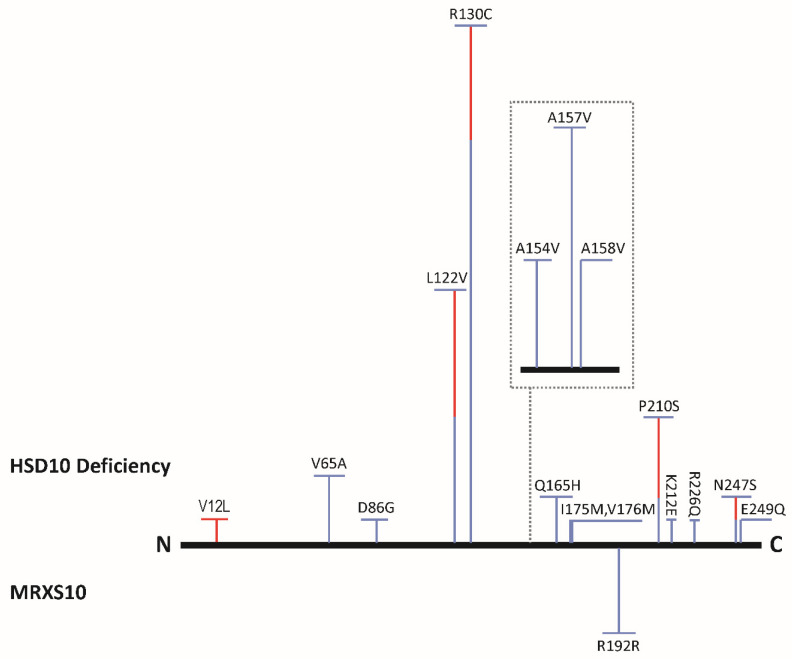
The missense mutations of 17β-HSD10, encoded by the *HSD17B10* gene, result in HSD10 deficiency, whereas a silent mutation causes mental disability, X-linked syndromic 10 (MRXS10). Female cases are indicated by a red vertical bar, whereas male cases are in blue. The bar height is approximately proportional to the number of cases. The contents shown in a dotted square were expanded by approximately 6x. The N and C represent the N- and C-terminals of 17β-HSD10, respectively. (Updated from Figure 5 of Ref. [3]).

The rediscovery of ERAB/ABAD in mitochondria was published as a *Science* report [33], which cited none of the previously reported literature on 17β-HSD10/SCHAD [1,5,8,9,10,11]. Most patients with this mitochondrial disease suffer from infantile neurodegeneration [36,37,38,39,40,41,42,43,44,45,46,47,48,49,50,51,52,53,54,55,56,57,58,59,60]. There are at least sixteen reported and clinically related missense mutations in the *HSD17B10* gene, but approximately half of such patients bear a 388C-T transition, resulting in a HSD10 (pR130C) mutant (Figure 5).

### 5.2. Diagnosis of HSD10 Deficiency

**Figure 6:**
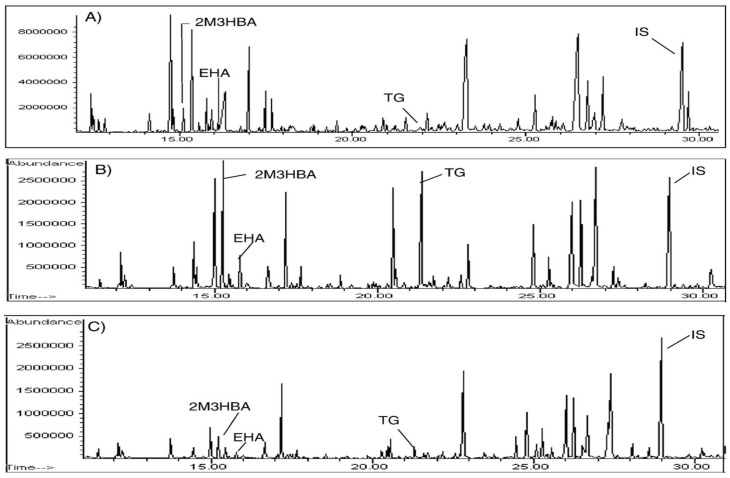
Urine organic acids were analyzed as trimethylsilyl (TMS) derivatives by gas chromatography–mass spectrometry. Urine organic acid profiles: (**A**) normal control; (**B**) patient with HSD10 deficiency; (**C**) patient with HSD10 deficiency under a low protein dietary regimen. 2MHBA, 2-methyl-3-hydroxybutyrc acid; EHA, 2-ethylhydracrylic acid; TG, tiglylglycine; IS, internal standard (reproduced from Figure 13.2 of Ref. [27]).

Patients suffering with this disease may have microcephaly but no dysmorphism or organomegaly. Clinical manifestations include epilepsy, choreoathetoid movements, ophthalmologic disorders and progressive neurodegeneration, psychomotor retardation or regression, hearing impairment and even cardiomyopathy, which causes some patients to experience a very short lifespan. This disease is an inborn error in isoleucine metabolism so that high levels of isoleucine metabolites—e.g., tiglylglycine, 2-methyl-3-hydroxybutyrate and 2-ethylhydracrylic acid (EHA), an intermediate in the R-pathway of isoleucine oxidation—would be detected in patients’ urine (see Figure 6). For the differential diagnosis of other isoleucine metabolism disorders, e.g., β-ketothiolase deficiency [57], a missense mutation must be identified in the *HSD17B10* gene by deoxynucleotide sequencing as hard evidence for this disease [36].

### 5.3. A Hot Spot in the HSD17B10 Gene

Approximately half of the cases of HSD10 deficiency are due to a C > T mutation in exon 4 of the *HSD17B10* gene; male patients with this mutation have a much more severe clinical phenotype than those bearing other mutations [3]. In order to answer the question as to why the p.R130C mutation is more frequently found in such patients, Rauschenberg et al. [56] suggested that “most other mutations in the *HSD17B10* gene are not observed because they are incompatible with life”. Such an explanation may be plausible, but it does not address the underlying molecular mechanism.

**Figure 7:**
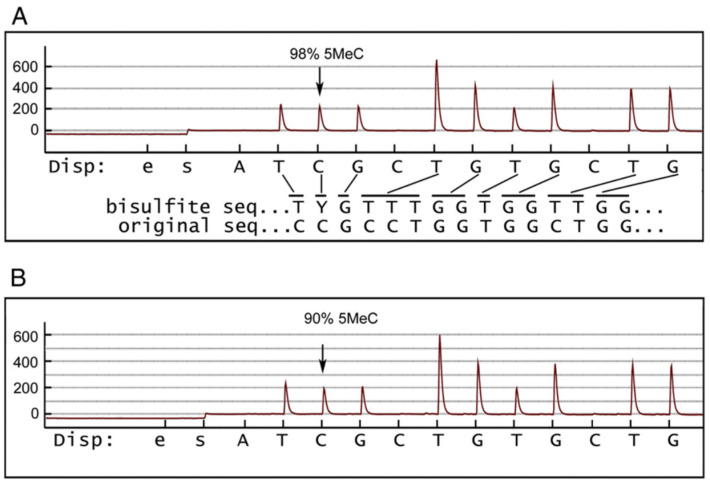
Methylation analysis of exon 4 of the *HSD17B10* gene. The bisulfite sequencing of chromosomal DNA from a normal male is displayed in (**A**), and that from a normal female in (**B**). The ordinate shows the relative light unit detected after dispensation of each nucleotide substrate. The abscissa shows the dispensation order (“e” and “s” are the controls). The “bisulfite” line shows the predicted sequence after modification. “Y” represents C or T. Lines connecting the dispensed nucleotide to the bisulfite sequence indicate how the light signal represents the modified sequence. The height of the signal is proportional to the number of nucleotides at that position [82] (reproduced from Figure 1 of Ref. [82]).

A methylation analysis of the *HSD17B10* gene [82,83] has later found a mutational hotspot, mCpG, at +2259 nucleotides from the initiation codon ATG of the HSD17B10 gene. The cytosine at that nucleotide position is >90% methylated in both active and inactive X chromosomes (Figure 7). Since the 5mC > T transition occurs approximately ten times more frequently than other transitions, this not only explains the high incidence of the p.R130C mutation in patients with HSD10 deficiency, but also suggests the possibility of a de novo mutation due to some environmental factor.

### 5.4. Sexuality Preference of This Disease Due to X-Inactivation

**Figure 8:**
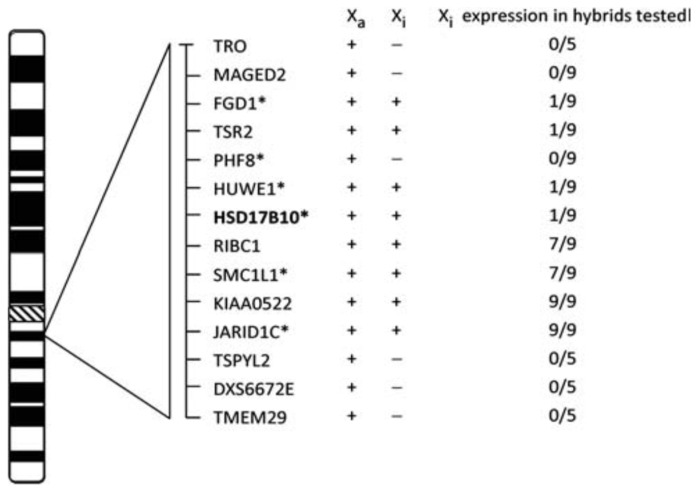
Escape of the HSD17B10 gene from X-chromosome inactivation. The expression of HSD17B10 transcripts from inactive X (Xi) hybrids. Samples scored as positive are expressed as at least >10% of the active X (Xa) levels; their number is shown as the numerator, whereas the total number of hybrids tested is shown as the denominator. An X-linked intellectual disability by gene mutation(s) bears an asterisk. Reproduced from Ref. [84].

HSD10 deficiency is an X-linked, inherited metabolic disease, and its onset occurs at 6–18 months after birth. Because the lifespan of most male patients is short and they do not reach their sexual maturity, a missense mutation at the *HSD17B10* gene in a child is usually inherited from his/her mother, a female carrier. In contrast to the hemizygosity of the X chromosome in males, females have two X chromosomes and one of them is inactive [84] due to X-inactivation (Figure 8). If the mutation happens in the inactive X chromosome, the mutated *HSD17B10* gene may not be able to generate HSD10 mutants. This mechanism explains why female carriers have relatively milder, or even no, symptoms.

### 5.5. Pathogenesis of HSD10 Deficiency

**Figure 9:**
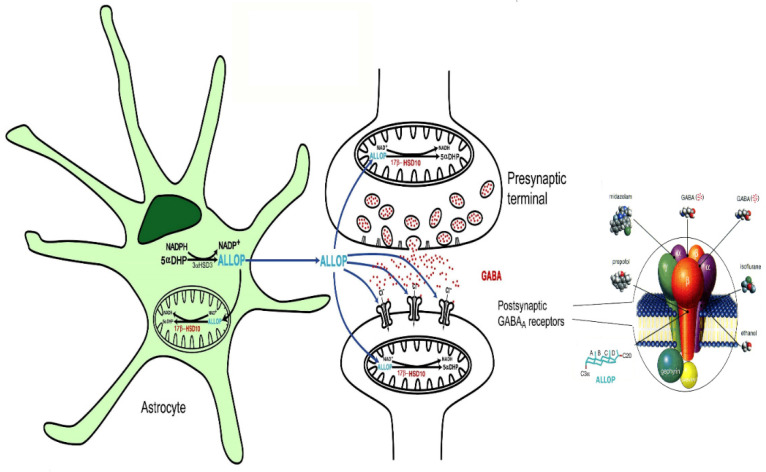
Roles of 17β-HSD10 in neurosteroid metabolism. 17β-HSD10 (red) is localized in the mitochondria and catalyzes the oxidation of allopregnanolone (ALLOP, blue), a positive modulator of GABAA receptors that potentiates GABA to increase the opening of Cl- channels. The postsynaptic GABAA receptor was magnified and is shown at the right side. ―• indicates the binding sites of individual modulators on the GABAA receptor [3]. Reproduced from Figure 2 of Ref. [3].

Although the accumulation of isoleucine and branched-chain fatty acid metabolites may not be completely benign [85,86], it does not cause neurological symptoms [36]. The excretion of isoleucine metabolite, e.g., tiglylglycine, 2-methyl-3-hydroxybutyrate and 2-ethylhydracrylic acid, is a clinical indicator [36]. However, the pathogenesis of HSD10 deficiency may lie in the disruption of other functions of 17β-HSD10, e.g., an imbalance of neurosteroid metabolism [2,3,13,36,82] and the impairing of mtRNA metabolism [49,52,56]. The interference of allopregnanolone metabolism may alter neuroexcitability [3] and lead to neurological disorders in patients (Figure 9).

## 6. Concluding Remarks

The reported stereo structures, intracellular localization and activities of ABAD/ERAB are indeed extremely distinct from those of 17β-HSD10. This is almost certainly due to the inability to reproduce any valid experiments concerning the localization and functions of ABAD/ERAB, notwithstanding their publication in highly regarded and widely read journals. It is no longer appropriate to confound the literature by employing the misnomer ABAD because it frustrates the progress of critical work in the studies of *HSD17B10*-gene-related disorders, including Alzheimer’s disease and intellectual disabilities. More importantly, clarification of the differences between ABAD and 17β-HSD10 will address the research integrity issues in this area. The removal of ABAD/ERAB contents from the entry OMIM300256, which is undoubtedly specific for the type 10 17β-hydroxysteroid dehydrogenase, appears to be essential for restoring a proper starting point in the treatment of *HSD17B10*-gene-related disorders including HSD10 deficiency, an inborn error in isoleucine and neurosteroid metabolism. Furthermore, the identification of more compounds to adjust 17beta-HSD10 or to regulate the HSD17B10 gene expression may promote a new approach to infantile or senile neurodegeneration in the future.

## Figures and Tables

**Table 1:** Generalized alcohol dehydrogenase and HAD activities of ABAD/ERAB *.

Substrate or Alcohol	K_m_ (mM)	V_max_ (Units/mg)	K_cat_ ^a^ (s^−1^)	Catalytic Efficiency, K_cat_/K_m_ (M^−1^s^−1^)
Reduction of S-acetoacetyl-CoA				
S-acetoacetyl-CoA	0.068 ± 0.020	430 ± 45	190	2.8 × 10^6^
Oxidation of alcohol substrate ^b^				
17β-Estradiol	0.014 ± 0.006	23 ± 3	10	7.4 × 10^5^
Methanol	No activity	No activity	No activity	No activity
Ethanol	1210 ± 260	2.2 ± 0.4	1.0	0.82
Isopropanol	150 ± 17	36 ± 2	16	110
n-Propanol	272 ± 62	4.2 ± 0.5	1.9	6.9
n-butanol	53 ± 6	9.0 ± 0.3	4.0	76
Isobutanol	56 ± 16	8.0 ± 0.7	3.6	64
n-Pentanol	18 ± 5	6.9 ± 0.4	3.1	170
(±)-2-Octanol	85 ± 17	245 ± 20	110	1300
(+)-2-Octanol	84 ± 16	102 ± 8	46	540
(−)-2-Octanol	43 ± 9.0	133 ± 23	60	1400
n-Decanol	14 ± 6.3	2.8 ± 0.5	1.3	90

* Reproduced from Table 13.1 of Ref. [27]. Data reported for a number of alcohols such as 2-octanol enantiomers and 17β-estradiol [19] were non-reproducible [4,7,21]. ^a^ Calculation based on 1 unit representing 1 μmol of product formed per min, and a molecular mass of the enzyme used for the calculation of *K*_cat_ was 26,926 Da because there was 1 active site per subunit. ^b^ Experiments were performed by incubating ERAB/HAD II with a range of concentrations of the indicated substrates in the presence of NAD^+^/NADH. Details of the experimental procedures are described in the text of Ref. [19].

**Table 2:** The preparation of assay mixtures to purportedly measure the *generalized* alcohol dehydrogenase activities of ABAD ^†^.

Tube	1	2	3	3′	4	5
(±)-2-Octanol	/	/	/	/	/	135 μL
(+)-2-Octanol	/	/	/	/	133 μL	/
(−)-2-Octanol	255 μL	333 μL	333 μL	333 μL	/	/
0.2 M Sodium phosphate (pH 8.8)	1100 μL	1100 μL	1100 μL	1100 μL	1100 μL	1100 μL
10 mM Sodium phosphate (pH 8.8)	300 μL	300 μL	300 μL	300 μL	300 μL	300 μL
75 mM NAD+	1000 μL	1000 μL	1000 μL	1000 μL	1000 μL	1000 μL
DMSO	/	/	100 μL	100 μL *	/	/
H_2_O	7345 μL	7267 μL	7167 μL	7167 μL	7467 μL	7465 μL

^†^ Reproduced from Table 1 of Ref. [7] * mixed with alcohol first and vortexed for 3 min; /, means “do not apply”.

## Abbreviations

ABAD: Aβ-peptide-binding alcohol dehydrogenase; AcAcCoA, acetoacetyl coenzyme A; AD, Alzheimer’s disease; CSF, cerebrospinal fluid; DMSO (Me_2_SO), dimethyl sulfoxide; ER, endoplasmic reticulum; ERAB, endoplasmic-reticulum-associated Aβ-binding protein; HAD, L-3-hydroxyacyl-CoA dehydrogenase; HAD2 or HADH II, 3-hydroxyacyl-CoA dehydrogenase type 2; HSD, hydroxysteroid dehydrogenase; 17β-HSD10, 17β-hydroxysteroid dehydrogenase type 10; HUGO, human genome organization; MHBD, 2-methyl-3-hydroxybutyryl-CoA dehydrogenase; MRXS10, mental disability, X-linked, syndromic 10; mtRNA, mitochondrial tRNA; PDB, protein data base; OMIM, Online Mendelian Inheritance in Man; SCHAD, short-chain 3-hydroxyacyl-CoA dehydrogenase.
